# Dissemination of the Knowledge of Dental Hygiene among the Masses
*Read before the Northern Ohio Dental Society, at Cleveland, O., May, 1889. Reprint from Ohio Journal of Dental Science, August, 1889.


**Published:** 1889-08

**Authors:** L. P. Bethel

**Affiliations:** Toledo, O.


ARTICLE III.

### DISSEMINATION OF THE KNOWLEDGE OF DENTAL HYGIENE AMONG THE MASSES.*

BY L. P. BETHEL, D. D. S., TOLEDO, O.

It is not difficult to advance a theory as to how the dissemination of knowledge of dental hygiene among the masses may be done, but it does seem difficult to obtain a means that will he practical, feasible, at the same time cover the entire ground.

Considerable has been said and written regarding this important subject, but as yet no definite plans in this direction have been adopted. With this matter in view, however, the American Dental Association have taken steps to inquire into the advisability of introducing the subject into the public schools. At their meeting in August, 1887, the following resolution was adopted:

*Resolved*, That Section II be directed to report at the next meeting of this association a plan or scheme for the introduction of a course of elementary instruction in dental histology, anatomy and hygiene into our public schools.

The secretary of that section communicated with superintendents of various cities in relation to the introduction of elementary instruction in these branches into the schools. The following questions being asked:

First. According to your jndgement, would you consider a course of lectures on the subjects mentioned, delivered at stated times by dentists or physicians, to be a benefit to the pupils?

Second. Do you think the body having control of education in your city would consider the matter favorably

*Read before the Northern Ohio Dental Society, at Cleveland, O., May, 1889. Reprint from Ohio Journal of Dental Science, August, 1889.

if it were indorsed and recommended by a professional body such as the American Dental Association?

Third. In what grade of the primary, grammar, or other schools would these lectures be most appropriate?

In his report at the last meeting of this Association in August, 1888, the secretary, Dr. Ottofy, said "that he received replies from thirty two cities, representing a population of 4,060,000 in 1888, and an enrolled school population of 750,000 in 1885. Of the thirty-two replies, twenty-four superintendents believed that the instruction would be beneficial to the pupils; the cities which they represent now having a total school population of 600,000. Only five superintendents believed that such a course of instruction would be of no value, and the school population of the cities which they represent is only 25,000.

To the question whether various Boards of education would permit the introduction of dental studies into the schools, twenty superintendents replied in the affirmative and nine superintendents did not believe that the school Boards would consider the subject favorably. A large majority are of the opinion that the high and grammar schools are most appropriate for the purpose."

Thus it will be seen that the majority heard from favor the introduction of such a course.

In States having laws requiring the study of anatomy, physiology, and hygiene, the introduction of suitable instruction in these branches would probably not be so difficult, as the more thoroughly prepared text could be added to the limited matter on dental hygiene already presented in the various text books, or be taken up in its stead.

The States at the present time making the study of anatomy, physiology and hygiene, with reference to the effects of alcohol and narcotics, compulsory in the public schools are: Maine, New Hampshire, Vermont, Connecticut, Rhode Island, New York, Pennsylvania, Delaware, Maryland, District of Columbia, West Virginia, and North Carolina.

The States and Territories requiring teachers to be examined in this branch and recommending it to be taught in the public schools yet not making it compulsory are: South Carolina, Florida, Kentucky, Alabama, Ohio, Michigan, Wisconsin, Minnesota, Iowa, Missouri, Dakota, Nebraska, Kansas, Montana, Wyoming, Colorado, New Mexico, Arizona, Utah, Idaho, Washington, Nevada, Oregon, and California. The remaining States and Territories, so far as we can learn, have made no requirements in this direction.

As to the proper grade or grades for this study should it not be introduced into all of them by means of oral and other instruction, together with suitable charts, models, books, and helps of various kinds according to the capabilities of the pupils? Let the instruction in the primary departments be oral, but in the higher grades let text-books be used in connection with oral instruction. The manner of presenting this study to be left entirely to the discretion of the teachers.

Lectures from various dentists or physicians may be given pupils or teachers at stated times, if thought best. This means would probably prove beneficial in at least stimulating a desire for the study, but should be carried on in such a manner as not to cause enmity among practitioners or strife to show a superiority of one dentist over another. The school Boards should arrange this matter themselves with due consideration and not at the solicitation of any particular practitioner, sect or sects. It might not be out of place for the acting dental society to instruct Boards regarding the selection of lecturers and remind them that, as a rule, the recognized dental society members keep thoroughly abreast of the rapid progress dentistry is making in all its departments. Again, the Boards might see an advantage in the selection of various practitioners to lecture or present papers bearing upon this subject. Special lectures to teachers alone or to teachers and advanced students by capable and non-resident dentists might be the means of keeping up or adding to the interest of the study.

It might be well for State or District Societies to appoint representatives to lecture in certain territories throughout each State, in school or other suitable halls, to teachers and students the public being invited to attend, and also lecture specially to teachers. Money for the purpose could be raised in various ways to be determined by official action. Teachers should be thoroughly interested in the study themselves and thoroughly instructed as to the great necessity of early and constant attention to the dental organs. Appropriate articles should be prepared to aid them in their instruction by chosen members of a dental society, bound together, in suitable book or pamphlet form, and furnished direct to teachers at cost. Text-books for students should be separately prepared in the same manner and also furnished at cost.

We can hardly expect the study to be made compulsory and whatever is accomplished in the school must be done through impressing superintendents, teachers, and school-boards of its great necessity.

While the introduction of this important study into the public schools is of great value, we cannot expect the best general results from this teaching of children alone. The parents must be educated as well and other means to accomplish this end must be devised. How then can we reach the parents?

*Through the Dental Office.*—Here an opportunity is afforded for imparting advice of a miscellaneous character to patients according to cases in hand and the surrounding circumstances.

To further the knowledge of dental hygiene, pamphlets could be prepared by dentists themselves, or suitable articles be prepared by society members, bound together in book or pamphlet form, issued by the society and furnished to dentists at cost, for distribution to patients. It might be well to have such works prepared and issued by State societies though under the supervision and approval of the American Dental Association. Thus a greater variety would be at command and an occasional change of books could be made, if desired, which might prove beneficial in several ways. Or let societies prepare copy for and print a regular publication, issued by the society and furnished dentists for distribution among their patients.

Another means of disseminating this knowledge is:

*Through Physicians.*—Physicians have, perhaps, a better opportunity to advise the general public, especially regarding the teeth of children, than do dentists. When called to a case they almost invariably note the condition of the tongue and other portions of the oral cavity in making up their diagnosis. If interested in the teeth how easily they could note their general condition, as regards caries, deposits, irregularity and general appearance. If in need of the dentist's care, physicians should note the conditions and at an opportune time make a more thorough examination and advise the parents of the fact, recommending as speedy attention to the matter as possible, explaining how decayed teeth or vitiated deposits assist in the production of disease. If not heeded before the time of a subsequent visit the request should be renewed. Advice from physicians would, as a rule, be heeded more promptly than that of the dentist, as too many people get the false impression that he is trying to "work up his trade."

Here another question arises: What will influence physicians to make these observations? At the present stage of progress, physicians and dentists should be intimate, understand each other and work harmoniously together. But few medical schools have special lectures on the teeth and their diseases, and what information the student acquires on this subject is from the subject matter in the various text-books which is indeed very limited. Physicians in general do not fully realize the necessity of careful attention to these organs. There is so much in medicine of direct importance to them that they are apt to neglect this subject even though the necessity for it be clearly pointed out. Far too many have held and still hold to the opinion that the decayed and aching teeth "must come out," and the forceps are directly applied. They are not familiar with the advancement the dental profession has made in the past few years and the present and improved methods in dental practice. Those who have neglected this study should receive instruction from the dentist regarding diseased teeth, their management, and all the different operations performed and conditions favoring each operation, that they may be able to differentiate and give prudent advice concerning them. The dentists in turn should receive such instruction from physicians, or suitable text-books, as will enable them to recognize and correctly diagnose abnormal conditions of the mouth and associate parts that should receive the attention of the physician, and recommend their patients' attention to the same. In this way early caries, irregularity, etc., would receive attention from the dentist, early tumors, cancers, and other abnormal conditions receive attention from the physician that otherwise might be overlooked or neglected until disastrous results followed. This interchangeable work would prove profitable to both and vastly beneficial to the patients of each.

Another means is:

*Through the Press.*—Articles should be prepared for the press by competent dentists, selected by associations for the purpose, and furnished to dentists at cost. Arrangements can be easily made for publishing in various country and city papers without cost to the dentist. If people have, through other sources had their attention called to the necessity of caring for the teeth, these articles would be apt to attract particular attention and be read with more interest. Such articles should be carefully prepared, short, concise, definite and clear. A series of articles printed in some of our leading magazines would have their weight, and especially if presented through such mediums as the Chautauquan or appear in the regular Chautauqua course. The practical articles prepared can be bound in book or pamphlet form and these furnished at cost which would probably bring them within reach of all reading classes.

Another method is:

*Through Lectures.*—Suitable lectures given to the public by non-resident dentists as before stated, would be an aid. To be the most effective all these different means should be carried on at the same time and the public thoroughly aroused as to the necessity for this instruction. It seems as though State societies should oversee the work in their own States, for people of different sections of the country differ so in their tastes and habits, that it is necessary that those fully acquainted with their peculiarities should deal with them, realizing better just the manner in which the most good can be accomplished, and where and how to expend the greatest effort. The State societies may be under the general control of the American if need be. Let the American represent the head and the State societies the hands that they may work harmoniously together in solving this perplexed question and bringing about a much needed reform.

In conclusion let us summarize the various methods herein advocated for the dissemination of the knowledge of dental hygiene among the masses:

First. Through the schools.

Second. Through the dental office.

Third. Through physicians.

Fourth. Through the periodicals.

Fifth. Through books and pamphlets.

Sixth. Through lectures.

As enumerated we have endeavored to set forth means for the advancement of this subject, but have made no attempt to arrange as to the order. Perhaps preparatory lectures should precede the other means in some localities, or other arrangement be advisable, all of which may be determined at a subsequent period.

Whatever is done, however, must be by united effort and through various agencies, to accomplish the best results. Too little attention has been given the subject, and it is hoped that it will soon be taken up in a systematic manner by the profession, and the proper effort made for its thorough dissemination.

